# Somatic mutations in SF3B1 aberrant‐negative MDS‐RS most commonly involved in TP53 genes

**DOI:** 10.1111/jcmm.17350

**Published:** 2022-05-18

**Authors:** Xiao Li, Feng Xu, Ling‐Yun Wu, Juan Guo, Qi He, Zheng Zhang

**Affiliations:** ^1^ Department of Hematology Shanghai Jiao Tong University Affiliated Sixth People's Hospital Shanghai China

1

To the Editor,

In general, most of the MDS‐RS (myelodysplastic syndromes with ring sideroblasts) is characterized by SF3B1 (splicing factor 3b subunit 1) mutations; Based on genetics, MDS with SF3B1 mutation may be defined as another subset of MDS (in addition to MDS with only 5q‐) in order to accurately explain the pathogenesis and allow patients to benefit from targeted therapy.^1^ However, the relationship between the genotype (SF3B1 mutations) and phenotype (RS) in MDS‐RS is not completely clear. Except for patients with typical SF3B1‐mutated MDS‐RS, a few patients with SF3B1‐mutated MDS do not exhibit ring sideroblasts. Moreover, some patients with ≥15% RS do not harbour SF3B1 mutation.

Emerging data show that some of the downstream effects of SF3B1 mutation converge on common cellular processes and cause aberrant mitochondrial iron metabolism. Nevertheless, it is still unclear whether some non‐splicing factor genes participate in iron metabolism in MDS. Since 2012, the tumour suppressor TP53 family has been reported to play roles in iron metabolism in different disorders.^2^ Recent studies have shown that ferroptosis can be regulated by p53 as well as by the tumour‐associated mutant p53 and the related signalling pathways.^3^ Malcovati et al^4^ ever reported that TP53 mutation was the most common mutation in MDS‐RS without SF3B1 mutations (9 of 93 cases) in 2015, and these TP53 mutations showed some different characteristics on Exon locus and mutant styles when compared to ordinary TP53‐mutated MDS. Swoboda et al^5^ recently reported that over half of those with wild type of SF3B1 appeared TP53 mutations and poor survival in a total 218 MDS cases with ≥5% of RS. Thus, in this study, we aimed to describe and discuss the genetic features of Chinese MDS‐RS patients who do not harbour SF3B1 mutations.

Data from this study were recorded after obtaining informed consent in accordance with the Declaration of Helsinki, and this study was approved by hospital review boards of the Shanghai Jiao Tong University Affiliated Sixth People's Hospital. MDS‐RS was diagnosed according to the 2016 diagnostic criteria^6^ (including few patients with CMML as determined by the FAB standard^7^), based on the premise that when patients were diagnosed with MDS, RS were present in ≥15% of all erythroblasts in the bone marrow, or RS were present in ≥5% of all erythroblasts when SF3B1 mutations were present. The clinical/experimental and genetic (next‐generation target sequencing) data of the patients were thoroughly analysed to define the features of those who had MDS‐RS but did not harbour SF3B1 mutations.

Of the 1019 MDS patients included in this study, 131 patients (12.9%) were diagnosed with MDS‐RS. By a general analysis of 39 common MDS‐related somatic mutations (*ANKRD11*, *ASXL1*, *BCOR*, *CALR*, *CBL*, *CEBPA*, *DHX9*, *DNMT3A*, *ETV6*, *EZH2*, *FLT3*, *GATA2*, *IDH1*, *IDH2*, *ITIH3*, *JAK2*, *KIF20B*, *KIT*, *KRAS*, *MPL*, *NF1*, *NPM1*, *NRAS*, *PHF6*, *PTPN11*, *PTPRD*, *ROBO1*, *ROBO2*, *RUNX1*, *SETBP1*, *SF3B1*, *SRSF2*, *STAG2*, *TET2*, *TP53*, *U2AF1*, *UPF3A*, *WT1* and *ZRSR2*), sixty‐seven of the 131 MDS‐RS (51.1%) patients harboured SF3B1 mutations. This frequency was lower than previously reported frequencies. Twenty‐five patients carrying SF3B1 mutations but with <5% RS were not included in this study according to the 2016 criteria.

Of the remaining 64 patients without SF3B1 mutations, 7 harboured zero mutations; 9 harboured ASXL1 or RUNX1 mutations, respectively; and fewer numbers of patients harboured other mutations. Since only 7.1% (63 out of 888 patients) of total non‐RS MDS patients harboured TP53 mutations, these mutations were identified in 21 of the 64 RS patients without SF3B1 mutations (32.8%) (or 16.0% of the total 131 MDS‐RS patients). We next compared some clinical/experimental and genetic features among the MDS‐RS patients with SF3B1 mutations, MDS‐RS patients with TP53 mutations and non‐RS‐MDS patients with TP53 mutations to explore the role of TP53 mutations in MDS‐RS.

We can see from Table 1 that the TP53‐mutated MDS‐RS tended to be more similar to SF3B1‐mutated MDS‐RS in terms of age at onset and sex ratio (showing a trend of older age and more males than TP53‐mutated non‐RS‐MDS). There were more MDS‐MLD patients than MDS‐SLD patients compared with SF3B1‐mutated MDS‐RS patients. Although TP53 is never considered a typical genotype of MDS‐RS, the RS ratio in the erythroblasts of these patients was not lower than that in typical MDS‐RS patients with SF3B1 mutations (both >30%). Otherwise, these patients were more similar to patients with TP53‐mutated MDS without RS. Many more patients with TP53‐mutated MDS‐RS had an IPSS score ≥1.5, and many more patients carried complex chromosomes.

**TABLE 1:** Comparison among different MDS subsets

	MDS‐RS with SF3B1 mutations A	MDS‐RS with TP53 mutations B	MDS (without RS) with TP53 mutations C	*p* value A vs. B	*p* value A vs. C	*p* value B vs. C
Case number	67	21	63			
Median age (years) (range)	67 (34–89)	71 (55–81)	65 (18–84)	0.197	0.068	0.021
Sex (M:F)	2.2	2.5	1.3	0.810	0.174	0.246
WHO classification						
MDS‐RS (SLD) (*N*/%)	26 (38.8)	5 (23.8)	None	0.209		
MDS‐RS (MLD) (*N*/%)	27 (40.3)	10 (47.6)	None	0.553		
MDS‐RS (EB/CMML) (*N*/%)	14 (21.0)	6 (28.6)	None	0.464		
MDS‐SLD+MLD (*N*/%)			28 (44.4)			
≥MDS‐EB/CMML (*N*/%)			35 (55.6)			
Median RS % in erythroblasts	Mean 39.9% Median 42% (5%–87%)	Mean 39.8% Median 32% (15%–78%)	None	0.806		
IPSS scoring						
≤1.0 (*N*/%)	58/64 (90.6)^a^	7/21 (33.3)	20/63 (31.7)	<0.001	<0.001	0.893
≥1.5 (*N*/%)	6/64 (9.4)^a^	14/21 (66.7)	43/63 (68.3)			
Bi‐allelic TP53 alterations (*N*/%)^b^		11/21 (52.4)	21/63 (33.3)			0.120
Chromosome						
Normal (*N*/%)	35/64 (54.7)	2/21 (9.5)	8/63 (12.7)	<0.001	<0.001	0.697
Abnormal (*N*/%)	25/64 (39.1)^a^	4/21 (19.0)	17/63 (27.0)	0.093	0.148	0.467
Complex (*N*/%)	4/64 (6.2)^a^	15/21 (71.4)	38/63 (60.3)	<0.001	<0.001	0.361
With 5q‐/7q‐; ‐5、‐7	4/64 (6.2)^a^	13/21 (61.9)	37/63 (58.7)	<0.001	<0.001	0.797

^a^ Means the denominator changed from 67 to 64 because of 3 failure chromosome analysis.

^b^ VAF ≥ 60% were defined as bi‐allelic alterations.

In addition, Figure 1(A) shows that compared to that in non‐RS MDS, the peak frequency of TP53 mutations in MDS‐RS appeared to be located in exon 8 (seven of the 21 patients; 33.3% vs. 15.9% of controls) and never involved exons 3 and 4. Additionally, this group had fewer nonsense mutations (4.8% vs. 11.1%). Most of the missense mutations found in these patients were located outside of the p53 DNA‐binding site in the β‐sandwich region (13/21 vs. 27/63 patients, *p *= 0.025) when the TP53 secondary crystal structures were analysed. Figure 1(B) presents the top three co‐mutation frequencies as well as EZH2 and RUNX1 in different subsets. One patient with TP53‐mutated MDS‐RS evolved to SF3B1‐mutated MDS‐RS after the decitabine‐induced disappearance of the TP53 mutation (Figure 1C). It seemed to provide a functional link between TP53 and SF3B1 in maintaining RS morphology. Finally, Figure 1(D) shows that TP53‐mutated MDS‐RS patients had obviously shorter overall survival (median 14 months) than typical SF3B1‐mutated MDS‐RS patients (median 40 months, *p *= 0.002), but their median OS (14 months) was relatively longer than that of non‐RS‐MDS patients with TP53 mutations (Figure 1E) When the survival of those with bi‐allelic TP53 alterations in group B(11/21 cases) and C (22/60 cases) were compared (Figure 1E),no significant difference was defined. Bi‐allelic alterations seemed not caused poorer prognosis as Bernard previously reported.^8^

**FIGURE 1:**
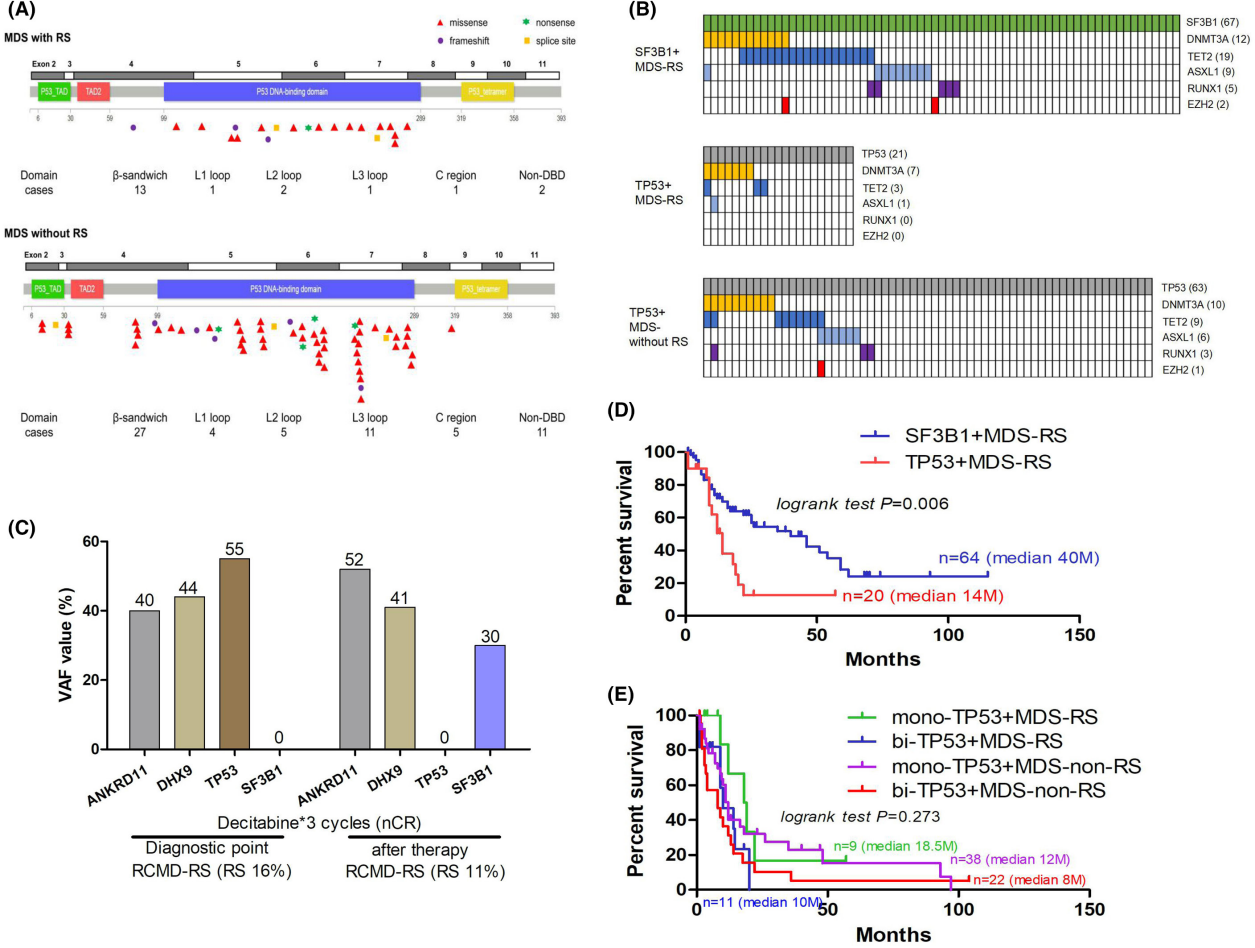
Feature of MDS‐RS without SF3B1. (A) Distribution of TP53 mutations in MDS with RS and without RS. (B) Co‐occurrence of TP53 with other mutations in different groups. (C) Change of gene mutations and RS before and after decitabine in a RCMD‐RS patient. (D) TP53‐mutated MDS‐RS patients had obviously shorter overall survival than SF3B1‐mutated MDS‐RS patients. (E) No difference was observed between MDS‐RS and non‐MDS‐RS patients with TP53 mutations even though grouped according to TP53 mutation status

Our study showed that some MDS‐RS patients who met the 2016 criteria did not carry SF3B1 mutations but did harbour other mutations affecting ASXL1, RUNX1 or TP53. To date, at least two papers have reported more frequent TP53 mutations in SF3B1 mutation‐negative MDS‐RS patients. Under the premise that SF3B1wt MDS‐RS must possess ≥15% of RS, Malcovati et al reported nine out of 93 patients without aberrant SF3B1‐mutated MDS‐RS had TP53 mutations.^5^ Our paper reported that 21 of 64 MDS‐RS patients who did not have SF3B1 mutations harboured TP53 mutations, and this number greatly exceeds those with ASXL1 and RUNX1 mutations (9 patients each). Given the findings in the last 10 years that indicated that the TP53 gene family participates in iron metabolism and ferroptosis development in some disorders, whether and how TP53 participates in MDS‐RS development warrants further investigation.

Our analysis showed that TP53‐mutated MDS‐RS patients have some features similar to those of SF3B1‐mutated MDS‐RS patients (older age and higher male ratio). They both had a mean RS number over >30% in their bone marrow (Table 1). However, TP53‐mutated MDS‐RS patients appeared to have more MDS‐MLD than MDS‐SLD, higher risk IPSS scores, more complex chromosomes, and a much shorter median overall survival (14 vs. 40 months) than SF3B1‐mutated MDS‐RS patients. They presented more features that were similar to those of TP53‐mutated non‐RS MDS patients in these prognosis‐related fields.

TP53‐mutated MDS‐RS patients harboured more TP53 mutations at the exon 8 locus, and the mutations found in these patients were easily located in the β‐sandwich region by secondary structure analysis. Given the importance of the loop structure rather than the β‐sandwich region for DNA binding,^9^ more mutations located in the β‐sandwich region may have decreased effects on TP53 function. Although the details are unclear and functional tests are lacking, TP53 mutations in MDS‐RS appeared not just an accompanied event. It seemed that the TP53 mutations in the MDS‐RS group serve two purposes. In addition to maintaining an aggressive clinical progression, these mutations also maintain the ring sideroblast phenotype.

Our results showed TET2 was the most common co‐mutations (19 of 67 patients; 28.4%) in the SF3B1‐mutated MDS‐RS subset, which was consistent with their excellent clinical progression and prognosis. TP53‐mutated MDS‐RS patients showed a 33.3% DNMT3A co‐mutation rate (seven out of the 21 patients), which is also consistent with their aggressive clinical characteristics. Figure 1(B) showed that almost none of ASXL1/EZH2 and RUNX1 co‐mutated with TP53 in group B, suggesting the poor prognosis of group B contributed from TP53 mutations themselves.

In summary, our findings provide some evidence that in addition to SF3B1, some other somatic gene mutations, especially TP53 mutations, could also be the genotype that causes the RS morphology in MDS, supporting the finding that the TP53 family participates in iron metabolism. Of course, more direct evidence is needed to validate this finding. Investigation of the patterns of mitochondrial iron metabolic in patients with TP53‐mutated MDS‐RS would be very helpful.

## AUTHOR CONTRIBUTIONS

**Xiao Li:** Conceptualization (lead); Funding acquisition (equal); Writing – original draft (lead); Writing – review & editing (lead). **feng xu:** Formal analysis (equal). **Lingyun Wu:** Formal analysis (equal). **Qi He:** Data curation (equal). **Juan Guo:** Data curation (equal); Software (equal). **Zheng Zhang:** Data curation (equal).

## CONFLICT OF INTEREST

The authors do not have any conflict of interest to declare.

## Funding information

This study was supported by the National Natural Science Foundation of China (grant nos. 81770120 and 81770122).

## Data Availability

The data can be available by contacting the corresponding author.
